# Theoretical and Experimental Study of Light-assisted Polymerization by Multimechanism Action

**DOI:** 10.1038/srep38473

**Published:** 2016-12-13

**Authors:** Hua Zhou, Dandan Song, Cheng Zhong, Guodong Ye

**Affiliations:** 1 Key Laboratory for Major Obstetric Diseases of Guangdong Province, The Third Affiliated Hospital of Guangzhou Medical University, Guangzhou 510150, People’s Republic of China; 2 Department of Chemistry, School of Pharmaceutical Sciences, Guangzhou Medical University, Guangzhou 511436, People’s Republic of China; 3 College of Chemistry and Molecular Sciences, Wuhan University, Wuhan 430072, People’s Republic of China

## Abstract

A novel bicomponent alkyd system was designed to decrease the usage of Cobalt-based drier due to its latent carcinogensis. The system was polymerized using a Cobalt-salt complex as an air-sensitive drier and an isopropylthioxanthone photoinitiator as a light-sensitive accelerator, as well as using irradiation in the form of visible light. The combined influence of the two additives on autoxidation was analyzed using real-time infrared spectroscopy. The results show that isopropylthioxanthone can increase the efficiency of hydrogen abstraction at the beginning of the curing reaction. A linear free energy relationship was used to theoretically predict the hydrogen abstraction ability of photoinitiators. Nanosecond laser flash photolysis was used to obtain the quenching rate constants between alkyd monomers and isopropylthioxanthone according to the Stern-Volmer equation. The thermodynamic data of transition state theory, such as the activation energy, were calculated by using quantum chemistry program. The reaction rate constant and the Wigner tunneling factor were predicted from the result of quantum chemistry. The vertical excitation energy obtained from the time-dependent density functional theory was used to explain the anomalous behavior of the photoinitiators. These theoretical results fit well with the experimental result of linear free energy relationship. On the basis of these observed results, an accelerated mechanism of the photoassisted autoxidation of alkyd resins was proposed.

More and more engineers use environmentally friendly additives to replace hazardous compounds in various formulations. Recently, cobalt organometallic complexes (Co salts, e.g., cobalt carboxylate) have received considerable attention due to their latent carcinogenic properties^1^. They are often used as a drier in the curing of alkyd monomers. Compared with other through and auxiliary driers, such as Ca, Zr, Al, and Ba-based catalysts, Co salts are still commonly used in commercial formulations. Therefore, much effort has been focused on the search of substitutes for cobalt complexes. However, although several substitutes, such as Mn or Fe-based complexes, are applied in industry, Co-based complexes remain widely used. The problem is that some cobalt alternatives are either inadequate in terms of activity or are strongly colored. Because an ideal solution is not yet available, the alternative approach is to minimize the usage of Co salts. For this purpose, a detailed understanding of the mechanism of autoxidation is necessary. The autoxidation reaction involves dehydrogenation from a methylene group between two double bonds in a lipid chain, yielding the pentadienyl radical –CH=CH–^**·**^CH–CH=CH–. The radical readily reacts with oxygen in air, leading to the intermediate alkyl hydroperoxide –C(OOH)–CH=CH–CH=CH–. In the autoxidation, the Co salts mainly catalyze the decomposition of hydroperoxides to generate alkoxy radicals (RO**·**), leading to polymerization (Haber and Weiss reaction). The details of the mechanism have not been clearly understood thus far; for specifics, the reader is referred to the original literature^2^,^3^,^4^. However, there is a general consensus that hydrogen abstraction (i.e., dehydrogenation) is the rate-determining step in this autoxidation. Thus, it seems worthwhile to innovate a formulation for increased efficiency of hydrogen abstraction. The bond dissociation energy (BDE) of *bis*-allylic C-H in lipids was reported to be approximately 50 kcal/mol lower than that of alkyl C-H (100 kcal/mol) and mono-allylic C-H (80 kcal/mol)^5^. So the alkyd monomer is active as a hydrogen donor. Taking a cue from photopolymerization, we suggested that hydrogen abstractor photoinitiators (Type II) can help to shorten the hydrogen abstraction process. 2-isopropylthioxanthone (ITX) is an efficient and common Type II photoinitiator and is recommended for use in formulations because it can absorb light energy in the visible region. ITX in the ground state can be initially excited to a high-energy singlet state (marked by 1) after being irradiated, as illustrated in Fig. 1. After that, it may convert to a more stable but less energetic triplet state (marked by 3) by intersystem crossing (marked by ISC). The utilization of sunlight is an important factor for outdoor jobs to accelerate the curing process. Thus, this approach was expected to result in the reduction of the amount of Co salts used because it can lower the barrier to the reaction. This is also called photoassisted autoxidation^6^.

The advantage of ITX is that it is applicable at low temperatures and is excited by sunlight, because its two maximum absorption peak of UV-*vis* spectrum in CH_2_Cl_2_ are at 258 nm (*ε* = 1.3 × 10^5^ M^−1^ cm^−1^) and 386 nm (*ε* = 6.1 × 10^4^ M^−1^ cm^−1^) respectively, tailing to approximately 420 nm. Without taking the monomer into consideration, the new bicomponent formulation was initiated by the air-sensitive drier (Co salt) mixed with the light-sensitive auxiliary accelerator (ITX) compared to the monocomponent system that is induced only by the Co salt^7^. The thrust of our research is to scientifically design a formulation, study its performance under kinetically controlled regimes, and model its performance under real conditions taking synergistic effects into account.

## Results

### Utilizing time-resolved IR to obtain photoassisted autoxidation profiles

Irradiation of bicomponent resins induced a continuous decrease of the absorption band at 3010 cm^−1^, which is assigned to the mono/biallelic H of non-conjugated double bonds^8^. The change in this band reflects the curing rate. The conversion (*α*) was calculated using the equation:


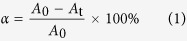


where *A*_0_ is the absorbance before irradiation and *A*_t_ is the absorbance at exposure time *t*. The polymerization rate, *R*_*p*_, is derived from the conversion and calculated using the equation:


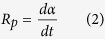


**Figure 2:**
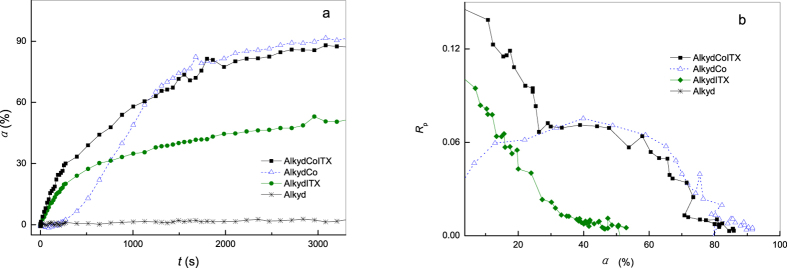
Polymerization profile based on the bicomponent formulation, light intensity: 40 mW·cm^−2^. (**a**) conversion (*α) vs.* exposure time (*t*); (**b**). Polymerization rate (*R*_p_) *vs.* conversion (*α*).

The result is shown in Fig. 2. Part a shows a plot of *α* versus *t* for four polymerizing systems. The reactivity of the monocomponent ITX (marked as AlkydITX) is obviously high; the conversion of the formulation is drastically increased before 500 s, at corresponding to a conversion of approximately 30%. After this, the conversion increases in a very smooth way as it approaches the final value (approximately 50%). The total conversion curve shows a characteristic skewed “L” shape. This indicates that ITX is most effective at the beginning of the reaction. The monocomponent Co salt (marked as AlkydCo) shows a pronounced induction period with a duration of 200 s in the early stage but particularly high activity in the final stage and the final conversion is over 80%. The shape of the conversion curve is approximately half-arched. For the bicomponent system (marked as AlkydCoITX), ITX significantly increases the rate of polymerization without an induction period.

In part b, the *R*_*p*_ is plotted as a function of *α*. The plots show two kinetic behaviors. The first is the plot of the AlkydITX resembles a diagonal-like line from the upper left to lower right; i.e., the reaction rate decreases dramatically with the conversion. The trace of the AlkydITX is unimodal and narrowed, which is similar to the behavior of autoacceleration in the UV-curing of acrylate^9^. The curve of the AlkydCo system shows the quasi-symmetrical form of a shallow arch. For the AlkydCoITX system, the *R*_*p*_ vs. *α* curve is similar to that of the AlkydITX system when *α* is less than 30%, but the curve of AlkydCoITX is higher. The increase is caused by the high mobility of primary radicals, apparently induced by ITX. But when *α* is over 30%, the behavior of AlkydCoITX is similar to that of AlkydCo. According to a report on the autoxidation by E. Bouwman, *pseudo*-first-order kinetics is a common phenomenon in autoxidation initiated by Cobalt salt drier^10^. So a conversion of 30% can be considered a borderline. The AlkydCoITX exhibits unexpected behavior of non-*pseudo*-first-order kinetics before the borderline because the two traces do not fit well together; it follows the mechanism of photopolymerization initiated by photoinitiator at this time. Then AlkydCoITX exhibits the usual characteristics of *pseudo*-first-order after the borderline as the two traces are coincident with each other.

### Utilizing the linear free energy relationship to predict the ability of hydrogen abstraction

It is well-known that spectroscopy, such as UV/Vis, phosphorescence and fluorescence, is a means of studying excited states in molecules. Phosphorescence spectroscopy is more applicable to investigate the hydrogen abstraction reaction occurring as a result of the triplet state of ITX. However, there are many difficulties hindering the broad application of phosphorescence probing techniques in industrial environments, since this technique is very sensitive to operating conditions. For example, in order to suppress non-radiative processes and increase quantum yields, one of the common procedures is to run luminescence measurements in frozen solutions and at cryogenic temperatures (77 K). To avoid these difficulties, we resorted to UV/Vis spectroscopy, to evaluate the ability of hydrogen abstraction of Type II photoinitiator. Our approach is based on a linear free energy relationship, which is intended to reflect the structure-property relationships of photoinitiators. The Kamlet-Taft expression has been found to be very successful for this purpose^11^. It is shown below:


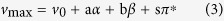


*v*_max_ and *v*_0_ are the solvent-dependent physicochemical properties in a given solvent or reference solvent, respectively. They may be the energy value (e.g. *E*_a_) or spectral data (e.g. λ_max_), etc. that we were interested in. π* is the dipolarity/polarizability, *α* describes the hydrogen-bond donating (HBD) ability, and *β* is the hydrogen-bond accepting (HBA) ability. The parameters *α, β*, and π* were obtained from the literature^12^, with a, b, and s, respectively, as their coefficients. The UV/Vis absorption spectra around the solvatochromic UV/Vis bands (the longest-wavelength band of the π-π* transition) of photoinitiators were measured in several common solvents at room temperature, as shown in Table 1. The results from multiple square analyses of the *ν*_max_ values of the photoinitiator measured in the solvents, with the Kamlet-Taft solvent parameters, are summarized in Table 2. THF is used as a reference (*α* = 0). For details, the reader is referred to Stefan Spange’s literature^13^. The correlations statistically provide a solid base to understand the manifold solvent effects on the varying reactivity of solute molecules. Next, we tried to predict the ability of hydrogen abstraction by applying this method. The rationale for this approach is as follows: in the alkyd resin, the active group initiating drying is the diallyl group from linoleic acid and its esters, as mentioned before. The BDE of *bis*-allylic C-H in lipids has been reported to be lower. In a sense, alkyd monomers and Type II photoinitiators can be identified as the hydrogen donors and as the hydrogen acceptors, respectively. Thus, the *β* can reflect the ability of hydrogen abstraction in a qualitative or quantitative way if the formation process of hydrogen-bonds is considered a part of the hydrogen transfer reaction. HBA and HBD are assumed to be the precursors of hydrogenated and dehydrogenated products. In general, the maximum peak in the UV spectrum is induced by the excitation from the ground state to the excited state. As a matter of fact, the broad spectrum is composed of many separate electronic transitions. However, the maximum absorption peak (**λ**_**max**_) was still selected for convenience. Since the reaction and property of organic compounds are often influenced by the solvent, in order to simulate the H-donor condition, many alcohol solvents were selected for this experiment. We hope the influence of solvent is almost like the H-donor, i.e. alkyd monomers. The parent compound of ITX, thioxanthone (TX), was also used to for comparison.

Comparing the two obtained equations, the b value for ITX (−0.590) is significantly lower than that for TX (−0.403). As stated previously, *β* represents the HBA, despite the fact that there is no hydrogen bond between them. The results show that ITX is predicted to perform worse than TX based on its higher b value. Two b values are negative numbers. This means both photoinitiators are characterized by hydrogen abstraction.

### Utilizing transient laser technology to detect quenching rate constants

**Figure 3:**
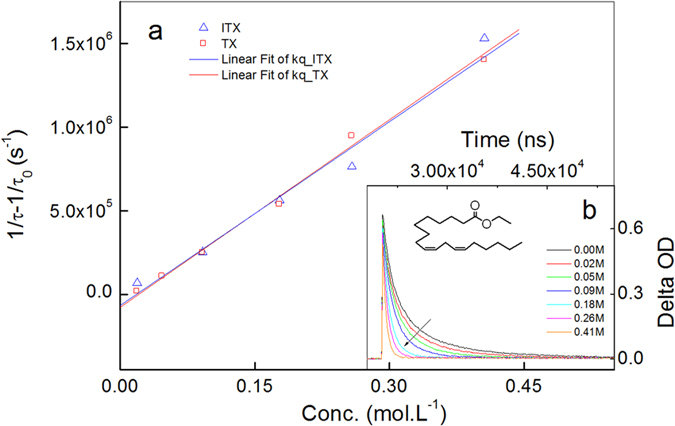
Results of the time-resolved laser flash photolysis experiment. (**a**) Stern-Volmer plot for the quenching of ^3^TX and ^3^TX in CH_2_Cl_2_. (**b**) Decay curve of ^3^TX quenched by ethyl linolenate as a descriptive example.

Quenching rate constants, *k*_q_, can be considered to reflect the interaction between an excited molecule (photoinitiator) and a quencher (alkyd monomer). Laser flash photolysis is used to measure *k*_*q*_ in order to investigate the photochemical process of ^3^ITX/^3^TX with ethyl linoleate as a quencher. The typical approach is to carry out a study of the lifetimes of ^3^ITX/^3^TX in the presence and absence of monomers. The temporal behavior was recorded with the decay signal of the photoinitiator, *F*, versus time, *t*, after the end of the laser pulse. The lifetime (*τ*) will decrease in the presence of a quencher if dynamic quenching is the mechanism that involved. Then, a kinetic analysis yielded the quenching rate constants, based on the influence of the alkyd monomer concentration, *Q*, on the decay signal of a photoinitiator. The Stern-Volmer analysis in a dynamic quenching reaction was used to determine *k*_*q*_, as shown in Fig. 3a.


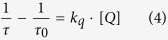


*Q* is the concentration of the quencher, *τ*_*0*_ and *τ* are the lifetimes of the photoinitiator in the absence and presence, respectively, of the quencher. The spectrum after the laser pulse shows the decay in the signal of the photoinitiator at 700 nm. Part b shows how the Stern-Volmer plot varies with the ethyl linolenate used as a quencher; the signal follows clean biexponential kinetics on the microsecond scale. The values of *k*_q_ from the Stern-Volmer calculation are 3.74 × 10^6^ M^−1^.s^−1^ for TX and 3.65 × 10^6^ M^−1^.s^−1^ for ITX respectively. The result shows that *k*_q_ was similar or higher to those observed previously for benzoyl, phosphinoyl, or ketyl radicals (10^5^–10^7^ M^−1^.s^−1^)^14^,^15^, indicating efficient interaction.

### Utilizing quantum chemistry to analyze the thermodynamic relation and kinetic process

**Figure 4:**
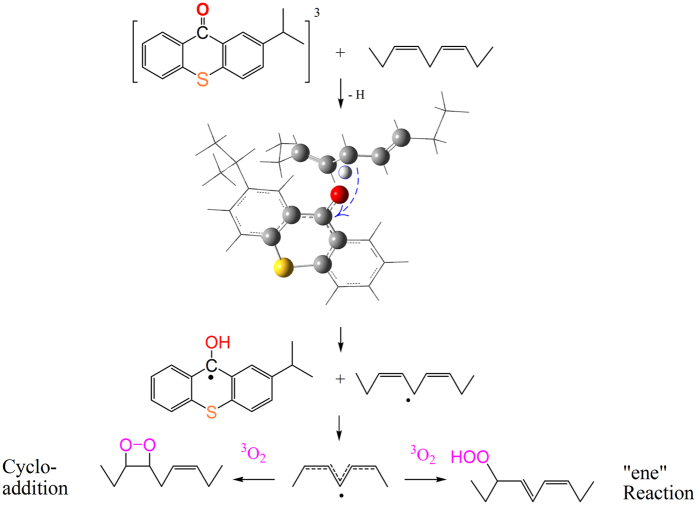
The proposed mechanism for photoassisted autoxidation.

In an attempt to probe the mechanism of the reaction, we began to explore the mechanistic details using quantum chemistry. Because of the lack of electron correlation, the energies from Hartree-Fock (HF) calculations are always greater than the exact energy. The disadvantage of semiempirical calculations is that the results can be parameter dependent and erratic. Thus fewer properties can be predicted reliably although they are much faster than *ab initio* calculations. For Density Functional Theory (DFT), the energy of a molecule can be determined from the electron density. This results in faster calculations than HF and gives more accurate results at the same time. The B3LYP hybrid functional with 6–31 G(d) basis set has been successfully used on a similar system contained a photoinitiator^16^,^17^. Therefore, we calculated the transition state (TS) of ITX compared to TX in the reactions with two compounds, methyl linoleate (MLO) and methyl oleate (MO) as model compounds of alkyd resins (MLOR and MOR are their corresponding radicals after dehydrogenation). Figure 4 shows the procedure, and the results of the calculations and their corresponding symbols are summarized in Table 3. The activation energies (*E*_*a*_) and reaction enthalpies (*ΔH*) of the four reactions and two thermoneutral reactions (MLO + MLOR and MO + MOR) were obtained from the results of optimization. Two thermoneutral reactions were used to calculate the Polanyi factor (*α*), which is obtained by the Evans-Polanyi relationship, and the Wigner tunneling factors is expressed by the following equations^18^. The kinetic results are shown in the same table.


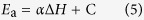



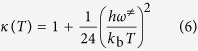


where *α* is Polanyi factor, *C* is the activation energy of the thermoneutral reaction (MLO + MLOR and MO + MOR) respectively. *κ(*T) is Wigner tunneling factor. *ω*^≠^ is the imaginary frequency of the transition state. *T* is the temperature. *k*_b_ and *h* are the Boltzmann constant and Planck constant, respectively.

We found that the structure of unsaturated monomer has a dramatic effect on the activation barrier in hydrogen abstraction reaction^19^. MO is known as a mono-ene. MLO is a special isolated diene because it has a methylene group between the two double bonds, thus MLO is also classified as a skipped diene. This is the shortest interval between two double bonds. The result shows that the hydrogen abstraction reaction had a lower potential energy when MLO was the reactant, and the corresponding rate constants were nearly two orders of magnitude bigger than that of MO. This suggests that dehydrogenation from a skipped diene was much easier. Comparing the data of two thermoneutral reactions between monomers, we found that the reaction of MLO had a lower activation barrier than MO too. However, the influence of photoinitiators was relatively small as their rate constants are of the same magnitude (e.g. ~10^−15^ for ITX). The electron-donating group in ITX cannot decrease the *E*_a_. Both *E*_a_ values of TX are slight smaller than that of ITX. The result of calculation is close to the previous prediction from linear free energy relationship.

The meaning of *α* is ambiguous, but it is represented as a transfer coefficient. However, according to the empirical results, it ranges from 0.2 to 0.6 when Δ*H* lies in the range of −40 to 0 kcal/mol for an exothermic atom transfer reaction^20^. On this basis, the results of MLO as H-donor fall within the scope of the *α* value while the results of MO do not accord with this rule.

The Wigner tunneling factor can be easily obtained and only requires the imaginary frequency of the transition state as the input parameter. This factor reflects the shape of the TS curve: smooth or steep. We found that the effect of the monomer on the tunneling factor is very obvious. In other words, the bigger the tunneling factor is, the steeper the TS curve and the higher the activation barrier. However, this was not relevant for the thermoneutral reaction. Compared with other hydrogen abstraction reactions (e.g., the value of a benzoyl radical reacting with a cyclohexadiene or a 2,5-heptadiene is 3.55 or 3.32)^18^, tunneling factors in triplet state systems are much smaller. The analysis is shown later in the discussion. From the above discussion, it is also suggested that the alkyd that contained a skipped diene group was a better monomer than the alkyd that had one double bond.

Based on the above results, the proposed mechanism for photooxidation is summarized in Fig. 4 (the effect of Co is ignored).

From studies of polymerizations initiated by the combination of a hydrogen abstractor with a cobalt complex, conclusions about the reaction mechanism of these autoxidations can be drawn, especially the role of the photoinitiator: ITX is excited to ^1^ITX, then ^3^ITX after irradiation, which abstracts a *bis*-allylic hydrogen atom from the polyunsaturated lipids, and pentadienyl radicals are yielded. These pentadienyl radicals react with oxygen, and then alkyl hydroperoxides are generated rapidly. This approach is represented as free-radical-promoted autoxidation. This appears to reduce the threshold for the Co salts so that it reacts more easily with the alkyd monomer. The curing reaction quickly passes around the hydrogen abstraction stage, which is considered a powerful obstacle in autoxidation.

## Discussion

As shown by the above results, ITX does not exhibit excellent properties compared to TX. The different reactivity of ITX and TX can be explained according to their excited energies. Theoretical results of time-dependent density functional theory (TD-DFT) showed that the vertical excitation energy of the singlet state of TX (85.65 kcal/mol) is slightly lower than that of ITX (86.96 kcal/mol). As can be see from the Jablonski diagram, the photoinitiator molecule was excited to a singlet state through vertical transition when exposed to light. The singlet state may convert by intersystem crossing to an excited triplet state. The hydrogen abstraction reaction occurs in the stage of triplet state. As can be seen from Table 3, the smaller activation energy and the smaller vertical excited energy indicated the TX would be easier to react with a skipped diene. These theoretical results fit well with the experimental result of linear free energy relationship.

In our earlier study, the tunneling factor was found to be above three in the ground state because of the huge imaginary frequency, occurring most often in thermoneutral reactions and hydrogen transfer induced by radicals. The values in the triplet state are lower than those in the ground state and have a pronounced impact on the reactivity. We believe that ITX in the triplet state acts like a biradicaloid compound. The amount of vibrational energy is in excess of that required to decrease the repulsion of another single electron in the molecule when the hydrogen atom gets closer to the excited ITX. Because the Wigner tunneling method uses the shape of V_MEP_ at the saddle point to estimate the tunneling correction, the marked discrepancy means that the shapes of V_MEP_ on the reactant side are not very similar. Near the saddle point, the stretch vibrations may decrease markedly; the adiabatic potential curve is expected to be even flatter near the saddle point. The harmonic turning points extend just past the lower contour and are very close to the concave side in the triplet state, indicating that the potential for this mode is quite harmonic. This observation precludes the reaction-path curvature because the skew angle is similar for the two states because the donor and the acceptor have the same mass-weighted average.

## Methods

### Materials

Alkyd (Num. 102–537, density = 0.94 g/ml) and Co salt (D645, ICI, DURHAM COBALT 10, containing cobalt carboxylate, symbol: Co) were obtained from Akzo Nobel(Netherland), and 2-isopropylthioxanthone (ITX) and the corresponding parent compound thioxanthone (TX) were obtained from BASF Co. (Germany). Ethyl oleate, ethyl linoleate, and ethyl linolenate were obtained from Aldrich Co. (USA).

### Measurements

The kinetic profiles of the simulated daylight-induced reactions were studied by real-time FTIR (RT-FTIR). Spectra were recorded on a modified Nicolet 6700, and the signal was detected using a DTGS and recorded. The light source used for irradiation was a Xenon spot light source with a shutter (Hamamatsu L9566). The light intensity was measured by a UV-radiometer from Photoeletric Instrument Factory (China). Light intensity was 40 mW·cm^−2^. The irradiation was controlled by the shutter. The infrared spectrometer recorded the IR signal when the shutter was turned off. The shutter (on/off) was triggered by the infrared spectrometer according to macro command. Since the whole sampling time (<2 min) is a very small proportion of the total reaction time (>1 hour), the IR signal was not influenced by the simulated light. A filter was used to cut off light below 400 nm and to achieve a spectral window of 400–700 nm. The rate of the reaction was monitored at 3010 cm^−1^, i.e., the *cis* H-C=CH stretching vibration. The initial IR absorption of the sample at 3010 cm^−1^ was approximately 0.12 ± 0.02.

UV/Vis spectra were obtained on a BECKMAN DU 640 instrument with a resolution of 0.1 nm and a cell length of 1 mm. Various amounts of monomers were dissolved in dichloromethane saturated with argon in the presence of ITX. The transient decay of ^3^ITX was detected by nanosecond laser flash photolysis at 700 nm, obtained from 355 nm laser excitation. The laser source was a Q-switched nanosecond Nd/YAG laser with the energy reduced down to 10 mJ pulse^−1^ (Power-lite 9010, Continuum). The transient absorption analysis system was LP900 (Edinburgh Instruments), equipped with a 450-W pulsed Xe arc lamp, a Czerny-Turner monochromator, a fast photomultiplier, and a transient digitizer. When the decay time is plotted *vs*. the concentration of monomers, *k*_q_ can be obtained from the slope of the linear regression line through the above points.

Calculations were carried out on fully optimized structures of reactants and products at the B3LYP/6–31 G(d) level by using Gaussian 09^21^ (see supplementary information). In order to ensure that the geometry was already converged to an energy minimum without any imaginary frequencies, we also calculated the frequency using the optimized geometry as a starting input file under the same basis set and functional. The reaction enthalpy (*ΔH*) for hydrogen abstraction was calculated as the energy difference between the products and the reactants (298 K). The rate constants and Wigner tunneling factors of the hydrogen abstraction reactions were computed using the Kinetic and Statistical Thermodynamical Package (KiSThelP), which is a cross-platform, free and open-source program to predict thermodynamic properties and rate constants from quantum chemistry results^22^. It was written in the programming language Java and can run a code on many different operating systems. All the computations were performed in the gas phase. M062x functional with 6–31 g(d) basic set was selected to obtain the vertical excitation energies by TD-DFT, with the option to opt for closed-shell systems.

## Additional Information

**How to cite this article**: Zhou, H. *et al*. Theoretical and Experimental Study of Light-assisted Polymerization by Multimechanism Action. *Sci. Rep.* **6**, 38473; doi: 10.1038/srep38473 (2016).

**Publisher's note:** Springer Nature remains neutral with regard to jurisdictional claims in published maps and institutional affiliations.

## Supplementary Material

Supplementary Information

## Figures and Tables

**Figure 1:**
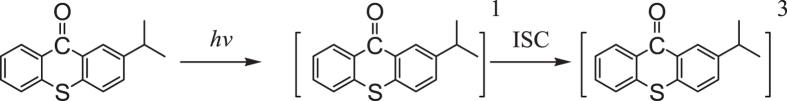
Excitation process of 2-isopropylthioxanthone (ITX) from ground state to triplet state. 1: singlet state, 3: triplet state, ISC: intersystem crossing.

**Table 1:** UV/Vis absorption maxima for photoinitiators in several solvents of different polarity and hydrogen bond ability.

Solvent	*v*_max_ ITX	*v*_max_ TX	α	*β*	π*
	(cm^−1^)	(cm^−1^)			
1-butanol	26.16	26.46	0.79	0.84	0.40
ethanol	26.15	26.50	0.86	0.75	0.54
methanol	26.11	26.47	0.98	0.66	0.60
isopropanol	26.16	26.53	0.76	0.84	0.48
THF	26.10	26.45	0.00	0.55	0.58
acetone	26.16	26.46	0.08	0.43	0.71
ethanediol	25.86	26.23	0.90	0.52	0.92

**Table 2:** Values of the solvent-independent correlation coefficients according to the Kamlet-Taft equation.

photoinitiator	*v*_0_	a	b	s	R^2^	Adjusted R^2^
TX	27.152	0.0375	−0.403	−0.78	0.762	0.525
ITX	27.048	0.0502	−0.590	−0.98	0.832	0.665

*ITX is the abbreviation of 2-isopropylthioxanthone, and TX is the abbreviation of thioxanthone.

**Table 3:** The result of quantum chemistry of the four reactions (MLO + TX/ITX and MO + TX/ITX) and two thermoneutral reactions (MLO + MLOR and MO + MOR).

	*E*_*a*_	Δ*H*	Δ*G*	*α*	ν	*κ*(T)	*k*
	kcal.mol^−1^	kcal.mol^−1^	kcal.mol^−1^		cm^−1^		cm^3^.molec^−1^.s^−1^
ITX + MLO	11.2	−25.8	−11.2	0.53	−331.18	1.11	1.62 × 10^−15^
ITX + MO	14.0	−13.7	−14.0	1.36	−857.17	1.71	2.47 × 10^−17^
TX + MLO	10.5	−26.8	−10.5	0.53	−228.95	1.05	5.42 × 10^−15^
TX + MO	13.2	−14.6	−13.2	1.34	−719.38	1.50	8.23 × 10^−17^
MLO + MLOR	24.8	0	0	—	−1763.55	4.02	3.01 × 10^−29^
MO + MOR	32.7	0	0	—	−1763.07	4.02	1.52 × 10^−30^

*ITX: 2-isopropylthioxanthone; TX: thioxanthone; MLO: methyl linoleate; MLOR: the dehydrogenated radical of methyl linoleate; MO: methyl oleate; MOR: the dehydrogenated radical of methyl oleate; *E*_a_: activation energy; Δ*H*: reaction enthalpy; *ν*: imaginary frequency; *α*: Polanyi factor; *κ*(T): tunneling factor; *k*: rate constant.
