# Supplementary material for: Theoretical and Experimental Study of Light-assisted Polymerization by Multimechanism Action
Source: Sci Rep. 2016 Dec 13;6:38473. doi: 10.1038/srep38473 (PMC5153847; doi:10.1038/srep38473)
Supplement: Supplementary Information [file srep38473-s1.pdf]

Theoretical and Experimental Study of Light-assisted Polymerization by Multimechanism Action

Hua ZHOU [1 #], Dandan SONG [2 #], Cheng ZHONG [3], Guodong YE [2 *]

1 Key Laboratory for Major Obstetric Diseases of Guangdong Province, The Third Affiliated Hospital of Guangzhou Medical University, Guangzhou 510150, People's Republic of China

2 Department of Chemistry, School of Pharmaceutical Sciences, Guangzhou Medical University, Guangzhou 511436, People's Republic of China

3 College of Chemistry and Molecular Sciences, Wuhan University, Wuhan 430072, People's Republic of China

* *Corresponding author.* Guodong YE *E-mail: gzhygd@gzhmu.edu.cn. Tel.: +86 13229494539. Fax: +86 2037103263.*

\# *These authors contributed to the work equally and should be regarded as co-first authors*

Supporting information

B3LYP/6-31g(d)

1 Triplet ITX

Center Number	Atomic Number	Atomic Type	X	Y	Z
1	6	0	4.587004	-1.235172	0.000112
2	6	0	4.910185	0.133373	0.000087
3	6	0	3.882657	1.070883	0.000086
4	6	0	2.547721	0.644627	0.000108
5	6	0	2.194436	-0.738730	0.000141
6	6	0	3.266989	-1.660536	0.000140
7	6	0	0.827967	-1.230388	0.000183
8	16	0	1.329874	1.912895	0.000043
9	6	0	-0.211147	1.093364	0.000116
10	6	0	-0.306256	-0.344664	0.000143

(Coordinates in Angstroms)

11	8	0	0.627024	-2.511984	0.000012
12	6	0	-1.618348	-0.881769	0.000139
13	6	0	-2.755754	-0.087549	0.000116
14	6	0	-1.357118	1.903026	0.000099
15	6	0	-2.618308	1.329868	0.000101
16	6	0	-4.133772	-0.725912	0.000112
17	6	0	-4.927909	-0.364385	1.271226
18	6	0	-4.927879	-0.364427	-1.271033
19	1	0	5.384238	-1.974063	0.000114
20	1	0	5.945545	0.458729	0.000069
21	1	0	4.108601	2.134313	0.000065
22	1	0	3.010318	-2.713325	0.000168
23	1	0	-1.697099	-1.963430	0.000159
24	1	0	-1.248383	2.984919	0.000084
25	1	0	-3.497019	1.966357	0.000090
26	1	0	-3.984777	-1.812975	0.000132
27	1	0	-5.892554	-0.885061	1.276444
28	1	0	-4.380591	-0.650860	2.175506
29	1	0	-5.130341	0.711656	1.326668
30	1	0	-5.892525	-0.885101	-1.276256
31	1	0	-4.380541	-0.650934	-2.175291
32	1	0	-5.130308	0.711613	-1.326517

Energy：

Zero-point correction= 0.252551 (Hartree/Particle)

Thermal correction to Energy= 0.268335

Thermal correction to Enthalpy= 0.269280

Thermal correction to Gibbs Free Energy= 0.207886

Sum of electronic and zero-point Energies= -1091.222614

Sum of electronic and thermal Energies= -1091.206830

Sum of electronic and thermal Enthalpies= -1091.205886

Sum of electronic and thermal Free Energies= -1091.267279

2 Triplet TX

Center Number	Atomic Number	Atomic Type	Coordinates (Angstroms) X	Y	Z

1	6	0	-3.706849	0.768998	0.000248
2	6	0	-3.812398	-0.637636	0.000311
3	6	0	-2.652113	-1.401342	0.000231
4	6	0	-1.399038	-0.771993	0.000091
5	6	0	-1.265805	0.656555	0.000022
6	6	0	-2.474408	1.398280	0.000108
7	6	0	-0.000037	1.349157	-0.000121
8	16	0	0.000063	-1.827409	0.000011
9	6	0	1.399095	-0.771900	-0.000038
10	6	0	1.265769	0.656639	-0.000093
11	8	0	-0.000067	2.652326	0.000070
12	6	0	2.474319	1.398447	-0.000116
13	6	0	3.706802	0.769248	-0.000090
14	6	0	2.652212	-1.401163	-0.000014
15	6	0	3.812446	-0.637379	-0.000039
16	1	0	-4.611402	1.371822	0.000307
17	1	0	-4.784593	-1.119549	0.000421
18	1	0	-2.707963	-2.487059	0.000278
19	1	0	-2.388885	2.478319	0.000051
20	1	0	2.388723	2.478480	-0.000163
21	1	0	2.708135	-2.486876	0.000026
22	1	0	4.784675	-1.119225	-0.000019
23	1	0	4.611315	1.372133	-0.000112

Energy:

Zero-point correction= 0.167760 (Hartree/Particle)

Thermal correction to Energy= 0.179092

Thermal correction to Enthalpy= 0.180036

Thermal correction to Gibbs Free Energy= 0.128794

Sum of electronic and zero-point Energies= -973.360853

Sum of electronic and thermal Energies= -973.349521

Sum of electronic and thermal Enthalpies= -973.348577

Sum of electronic and thermal Free Energies= -973.399819

3 ITX radical

Center Atomic Atomic Coordinates (Angstroms)

Number	Number	Type	X	Y	Z
1	6	0	-4.575205	-1.233510	0.205741
2	6	0	-4.890249	0.128069	0.285008
3	6	0	-3.872442	1.077159	0.191003
4	6	0	-2.545182	0.680595	0.018864
5	6	0	-2.201996	-0.696806	-0.046664
6	6	0	-3.259031	-1.639076	0.045098
7	6	0	-0.847100	-1.134648	-0.185038
8	16	0	-1.350836	1.971747	-0.212551
9	6	0	0.201820	1.125253	-0.101443
10	6	0	0.305752	-0.293132	-0.103955
11	8	0	-0.687898	-2.493240	-0.305844
12	6	0	1.614153	-0.842086	-0.011318
13	6	0	2.764808	-0.061527	0.029421
14	6	0	1.350905	1.914012	-0.049916
15	6	0	2.618249	1.335937	-0.000556
16	6	0	4.138129	-0.712314	0.123954
17	6	0	4.859772	-0.343781	1.434145
18	6	0	5.012154	-0.381486	-1.100686
19	1	0	-5.362125	-1.979712	0.273249
20	1	0	-5.918777	0.450832	0.417104
21	1	0	-4.109462	2.136643	0.244675
22	1	0	-3.011932	-2.692501	-0.011124
23	1	0	0.160481	-2.670868	-0.740176
24	1	0	1.730186	-1.919758	0.080642
25	1	0	1.252686	2.996693	-0.047662
26	1	0	3.491987	1.980347	0.031915
27	1	0	3.980518	-1.799715	0.131206
28	1	0	5.817907	-0.871803	1.507497
29	1	0	4.254861	-0.609296	2.307803
30	1	0	5.067963	0.731451	1.485880
31	1	0	5.973135	-0.906203	-1.041612
32	1	0	4.518194	-0.676761	-2.032830
33	1	0	5.223718	0.692663	-1.160961

Energy：

Zero-point correction= 0.266283 (Hartree/Particle)

Thermal correction to Energy= 0.282278
Thermal correction to Enthalpy= 0.283222
Thermal correction to Gibbs Free Energy= 0.221951
Sum of electronic and zero-point Energies= -1091.870988
Sum of electronic and thermal Energies= -1091.854992
Sum of electronic and thermal Enthalpies= -1091.854048
Sum of electronic and thermal Free Energies= -1091.915319

4 TX radical

Center Number	Atomic Number	Atomic Type	Coordinates (Angstroms) X	Y	Z
1	6	0	-3.709472	0.757877	-0.151079
2	6	0	-3.805292	-0.637763	-0.200349
3	6	0	-2.647296	-1.411318	-0.120238
4	6	0	-1.396527	-0.805369	0.008469
5	6	0	-1.275754	0.609924	0.041940
6	6	0	-2.471058	1.370616	-0.034469
7	6	0	-0.004011	1.258599	0.131433
8	16	0	-0.006169	-1.884588	0.231918
9	6	0	1.390363	-0.809395	0.044627
10	6	0	1.265308	0.609285	0.019619
11	8	0	-0.057813	2.627162	0.227603
12	6	0	2.464512	1.357041	-0.134445
13	6	0	3.706511	0.742625	-0.204752
14	6	0	2.643259	-1.417008	-0.038636
15	6	0	3.805112	-0.650855	-0.147586
16	1	0	-4.606733	1.367974	-0.208050
17	1	0	-4.772256	-1.122340	-0.298481
18	1	0	-2.713826	-2.495813	-0.150549
19	1	0	-2.393563	2.450869	-0.003269
20	1	0	0.760785	2.945314	0.638345
21	1	0	2.411474	2.436264	-0.249740
22	1	0	2.709233	-2.501822	-0.019489
23	1	0	4.772719	-1.140035	-0.205149
24	1	0	4.599451	1.350155	-0.322336

Energy：

Zero-point correction= 0.181500 (Hartree/Particle)

Thermal correction to Energy= 0.193042

Thermal correction to Enthalpy= 0.193986

Thermal correction to Gibbs Free Energy= 0.143053

Sum of electronic and zero-point Energies= -974.010750

Sum of electronic and thermal Energies= -973.999208

Sum of electronic and thermal Enthalpies= -973.998264

Sum of electronic and thermal Free Energies= -974.049197

5 Methyl oleate

Center Number	Atomic Number	Atomic Type	Coordinates (Angstroms) X	Y	Z
1	6	0	-9.089348	0.274287	-0.041119
2	6	0	-7.846272	-0.591912	-0.101272
3	6	0	-6.549800	0.202768	0.067118
4	6	0	-5.300769	-0.685840	0.022026
5	6	0	-3.993642	0.100911	0.183832
6	6	0	-2.739341	-0.781563	0.145363
7	6	0	-1.433396	0.007398	0.302757
8	6	0	-0.175776	-0.882200	0.264184
9	6	0	1.099842	-0.108910	0.462360
10	6	0	2.092789	-0.010871	-0.426098
11	6	0	3.368864	0.761867	-0.229211
12	6	0	4.625579	-0.129272	-0.263472
13	6	0	5.933317	0.659218	-0.118981
14	6	0	7.186213	-0.225821	-0.146185
15	6	0	8.496578	0.560270	-0.008362
16	6	0	9.748376	-0.326193	-0.031666
17	6	0	11.059885	0.458054	0.104445
18	6	0	12.304002	-0.435918	0.082790
19	8	0	-9.112315	1.471364	0.146743
20	8	0	-10.205169	-0.471247	-0.225062
21	1	0	-7.940200	-1.365535	0.673260
22	1	0	-7.859372	-1.134523	-1.056130

23	1	0	-6.492579	0.968139	-0.717023
24	1	0	-6.585178	0.750977	1.016731
25	1	0	-5.365494	-1.448967	0.812172
26	1	0	-5.276634	-1.237526	-0.929753
27	1	0	-3.927851	0.860960	-0.608448
28	1	0	-4.019511	0.655118	1.133596
29	1	0	-2.803714	-1.540002	0.939933
30	1	0	-2.715426	-1.338150	-0.803426
31	1	0	-1.359871	0.763977	-0.490679
32	1	0	-1.455197	0.562815	1.251890
33	1	0	-0.267494	-1.648480	1.050134
34	1	0	-0.136595	-1.421977	-0.691684
35	1	0	1.191720	0.422276	1.413166
36	1	0	2.000474	-0.542338	-1.376754
37	1	0	3.329540	1.305408	0.724471
38	1	0	3.461786	1.524936	-1.018092
39	1	0	4.643250	-0.694082	-1.207212
40	1	0	4.553740	-0.878069	0.537557
41	1	0	5.912037	1.229745	0.821524
42	1	0	5.997889	1.405897	-0.924584
43	1	0	7.204599	-0.799913	-1.084570
44	1	0	7.122264	-0.969435	0.662049
45	1	0	8.477458	1.136679	0.928628
46	1	0	8.561905	1.301935	-0.818461
47	1	0	9.767681	-0.903949	-0.967966
48	1	0	9.684164	-1.067142	0.779309
49	1	0	11.040372	1.035990	1.039482
50	1	0	11.125753	1.196892	-0.707022
51	1	0	13.223063	0.153007	0.182195
52	1	0	12.370063	-1.000480	-0.855548
53	1	0	12.283778	-1.162908	0.904122
54	6	0	-11.443530	0.254888	-0.180479
55	1	0	-12.224847	-0.486994	-0.349386
56	1	0	-11.574408	0.734923	0.793276
57	1	0	-11.468719	1.022883	-0.958387

Energy：

Zero-point correction= 0.524029 (Hartree/Particle)

Thermal correction to Energy= 0.551370
Thermal correction to Enthalpy= 0.552314
Thermal correction to Gibbs Free Energy= 0.458934
Sum of electronic and zero-point Energies= -895.653757
Sum of electronic and thermal Energies= -895.626417
Sum of electronic and thermal Enthalpies= -895.625472
Sum of electronic and thermal Free Energies= -895.718853

6 Methyl linoleate

Center Number	Atomic Number	Atomic Type	Coordinates (Angstroms) X	Y	Z
1	6	0	4.939758	-1.482527	-0.394380
2	6	0	3.881202	-1.456745	0.418990
3	6	0	2.443171	-1.329373	-0.027608
4	6	0	1.752701	-0.123742	0.566583
5	6	0	1.229564	0.886777	-0.132454
6	6	0	0.549974	2.097543	0.449689
7	6	0	6.374678	-1.596385	0.044041
8	1	0	4.776317	-1.403529	-1.472055
9	1	0	4.041098	-1.527869	1.497203
10	1	0	1.895147	-2.236779	0.276969
11	1	0	2.395189	-1.286226	-1.122679
12	1	0	1.701735	-0.093431	1.657240
13	1	0	1.286461	0.855021	-1.223742
14	1	0	1.124552	2.996196	0.178055
15	1	0	0.565366	2.039322	1.546611
16	1	0	6.420469	-1.720068	1.134505
17	1	0	6.820738	-2.505099	-0.390319
18	6	0	7.231298	-0.385798	-0.374471
19	1	0	7.158478	-0.250657	-1.463739
20	1	0	6.807811	0.523556	0.073944
21	6	0	8.706192	-0.520967	0.023400
22	1	0	9.121465	-1.436310	-0.424685
23	1	0	8.778622	-0.657811	1.112848
24	6	0	9.565683	0.680002	-0.392183
25	1	0	9.494425	0.816408	-1.480698

26	1	0	9.150783	1.594131	0.055791
27	6	0	11.037445	0.537064	0.008820
28	1	0	11.489511	-0.349834	-0.452299
29	1	0	11.624196	1.409229	-0.301650
30	1	0	11.144042	0.433893	1.095826
31	6	0	-0.901050	2.289509	-0.038821
32	1	0	-1.266203	3.263943	0.314620
33	1	0	-0.906406	2.343318	-1.137648
34	6	0	-1.865284	1.188984	0.420688
35	1	0	-1.489473	0.212521	0.085709
36	1	0	-1.869510	1.150107	1.520590
37	6	0	-3.298918	1.389038	-0.086594
38	1	0	-3.293362	1.429601	-1.185929
39	1	0	-3.673380	2.367240	0.249838
40	6	0	-4.269372	0.292735	0.371815
41	1	0	-3.894006	-0.686168	0.037770
42	1	0	-4.279247	0.253482	1.471461
43	6	0	-5.698186	0.496822	-0.146259
44	1	0	-5.697091	0.533588	-1.242732
45	1	0	-6.080216	1.470998	0.184068
46	6	0	-6.657912	-0.601044	0.316877
47	1	0	-6.313303	-1.591841	-0.009767
48	1	0	-6.700820	-0.655678	1.413028
49	6	0	-8.072405	-0.412125	-0.195499
50	8	0	-8.450990	0.479971	-0.923300
51	8	0	-8.887524	-1.389269	0.268922
52	6	0	-10.254914	-1.305159	-0.162133
53	1	0	-10.320504	-1.364999	-1.252024
54	1	0	-10.760544	-2.154458	0.298761
55	1	0	-10.705982	-0.365071	0.167157

Energy：

Zero-point correction= 0.500359 (Hartree/Particle)

Thermal correction to Energy= 0.527089

Thermal correction to Enthalpy= 0.528033

Thermal correction to Gibbs Free Energy= 0.435919

Sum of electronic and zero-point Energies= -894.444529

Sum of electronic and thermal Energies= -894.417799

Sum of electronic and thermal Enthalpies= -894.416855
Sum of electronic and thermal Free Energies= -894.508970

7 Methyl oleate radical

Center Number	Atomic Number	Atomic Type	Coordinates (Angstroms) X	Y	Z
1	6	0	8.445176	1.144042	0.173435
2	6	0	7.065154	1.062898	-0.449539
3	6	0	6.167736	0.013496	0.209500
4	6	0	4.775676	-0.059392	-0.429636
5	6	0	3.869037	-1.115395	0.215706
6	6	0	2.479204	-1.200001	-0.427398
7	6	0	1.574632	-2.271044	0.219181
8	6	0	0.238713	-2.403163	-0.446406
9	6	0	-0.983798	-2.203014	0.180210
10	6	0	-2.229404	-2.321612	-0.421334
11	6	0	-3.539715	-2.104031	0.271766
12	6	0	-4.375909	-0.957271	-0.336905
13	6	0	-5.745990	-0.789470	0.331906
14	6	0	-6.583748	0.348763	-0.265393
15	6	0	-7.961027	0.505152	0.392293
16	6	0	-8.800416	1.643117	-0.202754
17	6	0	-10.180841	1.793327	0.449448
18	6	0	-11.013375	2.930727	-0.151248
19	8	0	8.831394	0.506479	1.129048
20	8	0	9.217559	2.042348	-0.482981
21	1	0	6.612978	2.062679	-0.391718
22	1	0	7.190398	0.862615	-1.522046
23	1	0	6.657627	-0.966599	0.149907
24	1	0	6.078112	0.237442	1.279676
25	1	0	4.289505	0.925489	-0.364399
26	1	0	4.876596	-0.276272	-1.503679
27	1	0	4.358071	-2.098897	0.154823
28	1	0	3.762402	-0.895905	1.288252
29	1	0	1.979795	-0.223452	-0.363878
30	1	0	2.585858	-1.419372	-1.499742

31	1	0	2.108463	-3.235473	0.179420
32	1	0	1.439277	-2.040057	1.284716
33	1	0	0.240574	-2.670756	-1.504235
34	1	0	-0.964254	-1.935273	1.239699
35	1	0	-2.270149	-2.585270	-1.479344
36	1	0	-3.368657	-1.903762	1.338415
37	1	0	-4.143104	-3.026175	0.225186
38	1	0	-4.514548	-1.144092	-1.411698
39	1	0	-3.807349	-0.020260	-0.260957
40	1	0	-5.605834	-0.611083	1.408513
41	1	0	-6.306045	-1.733142	0.251904
42	1	0	-6.714771	0.175532	-1.343956
43	1	0	-6.028263	1.294052	-0.175895
44	1	0	-7.830769	0.676082	1.471366
45	1	0	-8.515865	-0.440498	0.300652
46	1	0	-8.927268	1.475359	-1.282872
47	1	0	-8.249207	2.590551	-0.106640
48	1	0	-10.054835	1.962078	1.528460
49	1	0	-10.731182	0.846533	0.352978
50	1	0	-11.991710	3.012557	0.336134
51	1	0	-11.187506	2.771436	-1.222671
52	1	0	-10.504113	3.896057	-0.040191
53	6	0	10.549209	2.200178	0.031557
54	1	0	11.024007	2.945528	-0.607492
55	1	0	10.523901	2.544518	1.069108
56	1	0	11.094354	1.253365	-0.012663

Energy：

Zero-point correction= 0.510420 (Hartree/Particle)

Thermal correction to Energy= 0.537645

Thermal correction to Enthalpy= 0.538590

Thermal correction to Gibbs Free Energy= 0.444851

Sum of electronic and zero-point Energies= -895.027263

Sum of electronic and thermal Energies= -895.000037

Sum of electronic and thermal Enthalpies= -894.999093

Sum of electronic and thermal Free Energies= -895.092831

8 Methyl linoleate radical

Center Number	Atomic Number	Atomic Type	Coordinates (Angstroms) X	Y	Z
1	6	0	4.939758	-1.482527	-0.394380
2	6	0	3.881202	-1.456745	0.418990
3	6	0	2.443171	-1.329373	-0.027608
4	6	0	1.752701	-0.123742	0.566583
5	6	0	1.229564	0.886777	-0.132454
6	6	0	0.549974	2.097543	0.449689
7	6	0	6.374678	-1.596385	0.044041
8	1	0	4.776317	-1.403529	-1.472055
9	1	0	4.041098	-1.527869	1.497203
10	1	0	1.895147	-2.236779	0.276969
11	1	0	2.395189	-1.286226	-1.122679
12	1	0	1.701735	-0.093431	1.657240
13	1	0	1.286461	0.855021	-1.223742
14	1	0	1.124552	2.996196	0.178055
15	1	0	0.565366	2.039322	1.546611
16	1	0	6.420469	-1.720068	1.134505
17	1	0	6.820738	-2.505099	-0.390319
18	6	0	7.231298	-0.385798	-0.374471
19	1	0	7.158478	-0.250657	-1.463739
20	1	0	6.807811	0.523556	0.073944
21	6	0	8.706192	-0.520967	0.023400
22	1	0	9.121465	-1.436310	-0.424685
23	1	0	8.778622	-0.657811	1.112848
24	6	0	9.565683	0.680002	-0.392183
25	1	0	9.494425	0.816408	-1.480698
26	1	0	9.150783	1.594131	0.055791
27	6	0	11.037445	0.537064	0.008820
28	1	0	11.489511	-0.349834	-0.452299
29	1	0	11.624196	1.409229	-0.301650
30	1	0	11.144042	0.433893	1.095826
31	6	0	-0.901050	2.289509	-0.038821
32	1	0	-1.266203	3.263943	0.314620
33	1	0	-0.906406	2.343318	-1.137648
34	6	0	-1.865284	1.188984	0.420688

35	1	0	-1.489473	0.212521	0.085709
36	1	0	-1.869510	1.150107	1.520590
37	6	0	-3.298918	1.389038	-0.086594
38	1	0	-3.293362	1.429601	-1.185929
39	1	0	-3.673380	2.367240	0.249838
40	6	0	-4.269372	0.292735	0.371815
41	1	0	-3.894006	-0.686168	0.037770
42	1	0	-4.279247	0.253482	1.471461
43	6	0	-5.698186	0.496822	-0.146259
44	1	0	-5.697091	0.533588	-1.242732
45	1	0	-6.080216	1.470998	0.184068
46	6	0	-6.657912	-0.601044	0.316877
47	1	0	-6.313303	-1.591841	-0.009767
48	1	0	-6.700820	-0.655678	1.413028
49	6	0	-8.072405	-0.412125	-0.195499
50	8	0	-8.450990	0.479971	-0.923300
51	8	0	-8.887524	-1.389269	0.268922
52	6	0	-10.254914	-1.305159	-0.162133
53	1	0	-10.320504	-1.364999	-1.252024
54	1	0	-10.760544	-2.154458	0.298761
55	1	0	-10.705982	-0.365071	0.167157

Energy：

Zero-point correction= 0.487132 (Hartree/Particle)

Thermal correction to Energy= 0.513664

Thermal correction to Enthalpy= 0.514608

Thermal correction to Gibbs Free Energy= 0.422484

Sum of electronic and zero-point Energies= -893.837336

Sum of electronic and thermal Energies= -893.810804

Sum of electronic and thermal Enthalpies= -893.809860

Sum of electronic and thermal Free Energies= -893.901983

9 Triplet ITX + methyl oleate : transition state

Center Number	Atomic Number	Atomic Type	Coordinates (Angstroms) X	Y	Z
1	6	0	3.807358	2.901121	-0.397311

2	6	0	2.837429	2.385145	-1.270827
3	6	0	2.468966	3.119883	-2.396094
4	6	0	3.058017	4.360253	-2.655632
5	6	0	4.047605	4.904598	-1.785130
6	6	0	4.398780	4.127498	-0.647807
7	6	0	4.694333	6.166167	-2.015901
8	16	0	2.469356	5.231217	-4.078028
9	6	0	3.499636	6.661374	-4.175526
10	6	0	4.460926	6.981461	-3.168785
11	8	0	5.595684	6.585698	-1.108992
12	6	0	5.222938	8.168550	-3.363923
13	6	0	5.056014	8.998206	-4.462197
14	6	0	3.322960	7.496102	-5.281414
15	6	0	4.084182	8.652619	-5.427686
16	6	0	5.916244	10.243746	-4.626492
17	6	0	6.819140	10.152650	-5.871702
18	6	0	5.069160	11.530055	-4.652981
19	1	0	4.101226	2.333104	0.481589
20	1	0	2.375511	1.421473	-1.077897
21	1	0	1.719374	2.729939	-3.080367
22	1	0	5.158250	4.529176	0.012469
23	1	0	5.973761	8.393594	-2.614207
24	1	0	2.587181	7.233468	-6.037798
25	1	0	3.930216	9.281038	-6.300241
26	1	0	6.572215	10.298068	-3.747577
27	1	0	7.474349	11.029504	-5.938478
28	1	0	7.448725	9.257134	-5.837901
29	1	0	6.225615	10.109755	-6.792795
30	1	0	5.713144	12.415579	-4.712313
31	1	0	4.452384	11.617617	-3.751382
32	1	0	4.397800	11.549729	-5.519766
33	6	0	2.794844	8.283735	9.257898
34	6	0	2.706406	8.920604	7.884528
35	6	0	3.306218	8.050585	6.778136
36	6	0	3.212468	8.706154	5.394931
37	6	0	3.806506	7.840895	4.275606
38	6	0	3.709614	8.490668	2.889405
39	6	0	4.308799	7.617862	1.771712

40	6	0	4.222886	8.265133	0.390200
41	6	0	2.905418	8.342494	-0.235877
42	6	0	2.513821	9.307174	-1.106453
43	6	0	1.178277	9.375651	-1.781037
44	6	0	0.380292	10.641635	-1.403122
45	6	0	-0.972680	10.739572	-2.119755
46	6	0	-1.771691	11.990547	-1.733048
47	6	0	-3.132099	12.093073	-2.435747
48	6	0	-3.930138	13.342065	-2.041106
49	6	0	-5.293992	13.445566	-2.736535
50	6	0	-6.084111	14.694952	-2.334311
51	8	0	3.308454	7.216202	9.513427
52	8	0	2.215606	9.073882	10.192884
53	1	0	1.650071	9.145634	7.685055
54	1	0	3.206899	9.897360	7.935557
55	1	0	4.353975	7.832959	7.019628
56	1	0	2.794858	7.079879	6.766987
57	1	0	2.159302	8.925579	5.163679
58	1	0	3.727153	9.678475	5.413999
59	1	0	4.860342	7.624665	4.503947
60	1	0	3.293918	6.868028	4.257888
61	1	0	2.657460	8.706567	2.655366
62	1	0	4.223904	9.462760	2.903867
63	1	0	5.359998	7.402715	2.001972
64	1	0	3.793495	6.647719	1.746131
65	1	0	4.777582	9.210203	0.325192
66	1	0	4.889947	7.524729	-0.318097
67	1	0	2.210755	7.528279	-0.019432
68	1	0	3.213149	10.113490	-1.334309
69	1	0	0.590535	8.480393	-1.540361
70	1	0	1.330021	9.369264	-2.871702
71	1	0	0.982647	11.531846	-1.634983
72	1	0	0.223402	10.653239	-0.315704
73	1	0	-1.568748	9.842987	-1.894539
74	1	0	-0.809924	10.730886	-3.207617
75	1	0	-1.176222	12.886774	-1.962153
76	1	0	-1.925272	11.999474	-0.643718
77	1	0	-3.726448	11.195624	-2.208371

78	1	0	-2.980133	12.087700	-3.525370
79	1	0	-3.337466	14.240206	-2.270936
80	1	0	-4.078725	13.348732	-0.950832
81	1	0	-5.885979	12.548159	-2.506717
82	1	0	-5.146196	13.440468	-3.825920
83	1	0	-7.052379	14.738651	-2.845962
84	1	0	-5.533407	15.610074	-2.584818
85	1	0	-6.276978	14.711322	-1.254476
86	6	0	2.246680	8.562444	11.534814
87	1	0	1.741686	9.311495	12.145743
88	1	0	1.724880	7.603384	11.594073
89	1	0	3.277799	8.424425	11.871582

Energy：

Zero-point correction= 0.773456 (Hartree/Particle)

Thermal correction to Energy= 0.817720

Thermal correction to Enthalpy= 0.818664

Thermal correction to Gibbs Free Energy= 0.683025

Sum of electronic and zero-point Energies= -1986.873434

Sum of electronic and thermal Energies= -1986.829170

Sum of electronic and thermal Enthalpies= -1986.828226

Sum of electronic and thermal Free Energies= -1986.963866

10 Triplet TX + methyl oleate : transition state

Center Number	Atomic Number	Atomic Type	Coordinates (Angstroms) X	Y	Z
1	6	0	3.942800	2.873221	-0.374421
2	6	0	2.967411	2.330068	-1.225195
3	6	0	2.550734	3.054183	-2.340563
4	6	0	3.097698	4.310753	-2.613167
5	6	0	4.092554	4.882194	-1.766368
6	6	0	4.492922	4.115859	-0.638083
7	6	0	4.697332	6.161261	-2.012149
8	16	0	2.449124	5.165773	-4.018987

9	6	0	3.432692	6.628528	-4.140516
10	6	0	4.411361	6.972055	-3.156581
11	8	0	5.610095	6.604701	-1.128666
12	6	0	5.134330	8.181913	-3.367431
13	6	0	4.890641	8.990882	-4.462354
14	6	0	3.193889	7.454763	-5.241005
15	6	0	3.912784	8.638134	-5.407534
16	1	0	4.273203	2.313822	0.496948
17	1	0	2.537833	1.353639	-1.022024
18	1	0	1.795844	2.643203	-3.006409
19	1	0	5.255711	4.539797	0.004134
20	1	0	5.900832	8.432146	-2.643596
21	1	0	2.444994	7.165104	-5.974128
22	1	0	3.722320	9.271022	-6.269159
23	6	0	2.776546	8.258731	9.248955
24	6	0	2.688858	8.899805	7.877483
25	6	0	3.296350	8.036551	6.769986
26	6	0	3.203031	8.696128	5.388665
27	6	0	3.804458	7.837490	4.268176
28	6	0	3.708433	8.491630	2.883992
29	6	0	4.314093	7.625340	1.764869
30	6	0	4.229971	8.278210	0.385524
31	6	0	2.912073	8.358488	-0.240920
32	6	0	2.524208	9.325642	-1.110558
33	6	0	1.190899	9.398087	-1.788480
34	6	0	0.395664	10.666310	-1.411998
35	6	0	-0.955684	10.767472	-2.131269
36	6	0	-1.752774	12.019952	-1.745453
37	6	0	-3.112070	12.125053	-2.449942
38	6	0	-3.908529	13.375247	-2.055908
39	6	0	-5.271471	13.481004	-2.752799
40	6	0	-6.060153	14.731447	-2.351047
41	8	0	3.293539	7.192256	9.502070
42	8	0	2.192159	9.043563	10.185164
43	1	0	1.631931	9.119967	7.675677
44	1	0	3.184071	9.878980	7.933204
45	1	0	4.344561	7.823646	7.013711
46	1	0	2.790137	7.063215	6.754281

47	1	0	2.149397	8.910974	5.155262
48	1	0	3.712737	9.670966	5.412232
49	1	0	4.858662	7.625553	4.498789
50	1	0	3.296587	6.862251	4.245698
51	1	0	2.655973	8.704005	2.648034
52	1	0	4.218598	9.465812	2.903181
53	1	0	5.365465	7.413294	1.996989
54	1	0	3.802637	6.653312	1.733879
55	1	0	4.781940	9.225438	0.327357
56	1	0	4.895324	7.547875	-0.323558
57	1	0	2.216217	7.544517	-0.027594
58	1	0	3.225407	10.130833	-1.335620
59	1	0	0.599747	8.504401	-1.550324
60	1	0	1.346967	9.392273	-2.878548
61	1	0	1.000838	11.554847	-1.642663
62	1	0	0.236499	10.678241	-0.324913
63	1	0	-1.554120	9.872088	-1.907565
64	1	0	-0.790821	10.758918	-3.218804
65	1	0	-1.155300	12.915072	-1.973604
66	1	0	-1.907822	12.028971	-0.656332
67	1	0	-3.708237	11.228544	-2.223620
68	1	0	-2.958692	12.119777	-3.539364
69	1	0	-3.314185	14.272490	-2.284899
70	1	0	-4.058301	13.381905	-0.965796
71	1	0	-5.865039	12.584412	-2.523881
72	1	0	-5.122503	13.476010	-3.842022
73	1	0	-7.027748	14.776790	-2.863818
74	1	0	-5.507762	15.645814	-2.600584
75	1	0	-6.254264	14.747722	-1.271434
76	6	0	2.221843	8.527734	11.525462
77	1	0	1.712674	9.272908	12.137660
78	1	0	1.703365	7.566609	11.580241
79	1	0	3.252641	8.392314	11.864247
80	1	0	5.467572	9.902490	-4.595589

Energy：

Zero-point correction= 0.688909 (Hartree/Particle)

Thermal correction to Energy= 0.728656

Thermal correction to Enthalpy= 0.729600
Thermal correction to Gibbs Free Energy= 0.604310
Sum of electronic and zero-point Energies= -1869.013066
Sum of electronic and thermal Energies= -1868.973319
Sum of electronic and thermal Enthalpies= -1868.972375
Sum of electronic and thermal Free Energies= -1869.097665

11 Triplet ITX + methyl linoleate : transition state

Center Number	Atomic Number	Atomic Type	Coordinates (Angstroms) X	Y	Z
1	6	0	-1.481000	-0.455301	-2.878139
2	6	0	-0.017307	-0.884921	-3.107625
3	6	0	0.968397	0.084677	-2.524601
4	6	0	1.898913	-0.213056	-1.597966
5	6	0	2.837790	0.763957	-0.999684
6	6	0	4.193407	0.265001	-0.656468
7	6	0	5.321321	0.993003	-0.720663
8	6	0	6.691806	0.499599	-0.356284
9	6	0	7.682537	0.528953	-1.537846
10	1	0	-1.659384	-0.352034	-1.800362
11	1	0	-1.634865	0.544345	-3.310749
12	1	0	0.147175	-1.883850	-2.682635
13	1	0	0.152801	-0.973871	-4.192603
14	1	0	0.903047	1.112605	-2.887593
15	1	0	1.961594	-1.237177	-1.226240
16	1	0	2.866997	1.710170	-1.553721
17	1	0	4.249700	-0.761146	-0.288989
18	1	0	5.261486	2.022791	-1.079494
19	1	0	6.625751	-0.520783	0.044011
20	1	0	7.098099	1.127147	0.452974
21	1	0	7.296344	-0.110247	-2.343539
22	1	0	7.725402	1.547792	-1.949439
23	1	0	2.350151	1.081339	0.035771
24	8	0	1.635025	1.472831	1.337478
25	6	0	0.732572	0.518798	1.574302
26	6	0	1.174573	-0.706726	2.182867

27	6	0	2.540737	-0.863363	2.543155
28	6	0	3.008363	-2.028915	3.126000
29	6	0	2.138355	-3.100875	3.384906
30	6	0	0.789800	-2.978203	3.058121
31	6	0	0.308585	-1.804920	2.469521
32	6	0	-1.698907	-0.144844	1.459580
33	6	0	-3.019659	0.187093	1.141529
34	6	0	-3.326254	1.434583	0.608196
35	6	0	-2.304160	2.384069	0.373837
36	6	0	-0.996997	2.047742	0.690868
37	6	0	-0.637161	0.787894	1.243707
38	6	0	-2.627689	3.752003	-0.209523
39	6	0	-3.545979	4.567581	0.720795
40	6	0	-3.227832	3.644904	-1.624414
41	1	0	3.201677	-0.025450	2.355421
42	1	0	4.058996	-2.109784	3.393298
43	1	0	2.505391	-4.014399	3.842771
44	1	0	0.102131	-3.795208	3.262740
45	1	0	-3.811853	-0.535908	1.320767
46	1	0	-4.360124	1.671765	0.374716
47	1	0	-0.191457	2.756586	0.534107
48	1	0	-1.678108	4.296589	-0.294719
49	1	0	-3.102634	4.677158	1.716302
50	1	0	-3.717533	5.569919	0.310387
51	1	0	-4.523742	4.085967	0.840856
52	1	0	-2.562605	3.089582	-2.294784
53	1	0	-4.195459	3.129245	-1.611858
54	1	0	-3.388872	4.642194	-2.050989
55	16	0	-1.415658	-1.765984	2.089027
56	6	0	-2.498211	-1.432449	-3.479898
57	1	0	-2.309476	-1.542281	-4.558531
58	1	0	-2.346183	-2.430193	-3.041794
59	6	0	-3.954177	-1.001145	-3.260634
60	1	0	-4.110295	-0.012203	-3.714818
61	1	0	-4.132441	-0.870900	-2.184065
62	6	0	-4.971037	-1.993062	-3.834590
63	1	0	-4.864575	-2.982081	-3.371965
64	1	0	-5.999989	-1.655457	-3.665083

65	1	0	-4.836061	-2.118751	-4.916072
66	6	0	9.095000	0.075587	-1.148355
67	1	0	9.474743	0.723197	-0.344088
68	1	0	9.046931	-0.938379	-0.724130
69	6	0	10.085438	0.086885	-2.319967
70	1	0	10.131613	1.099090	-2.747859
71	1	0	9.707950	-0.564414	-3.121752
72	6	0	11.499057	-0.361472	-1.927272
73	1	0	11.878402	0.293689	-1.128796
74	1	0	11.451862	-1.371423	-1.492943
75	6	0	12.483461	-0.357646	-3.103187
76	1	0	12.535746	0.645877	-3.543908
77	1	0	12.116432	-1.014894	-3.901183
78	6	0	13.890150	-0.800747	-2.696558
79	1	0	14.301547	-0.150901	-1.912610
80	1	0	13.877133	-1.809271	-2.260720
81	6	0	14.869608	-0.811594	-3.854225
82	8	0	14.608742	-0.543507	-5.007025
83	8	0	16.106587	-1.169727	-3.435113
84	6	0	17.112763	-1.216980	-4.459108
85	1	0	17.234895	-0.235516	-4.925396
86	1	0	18.031284	-1.517742	-3.953944
87	1	0	16.843797	-1.943260	-5.231006

Energy：

Zero-point correction= 0.750610 (Hartree/Particle)

Thermal correction to Energy= 0.794382

Thermal correction to Enthalpy= 0.795327

Thermal correction to Gibbs Free Energy= 0.661244

Sum of electronic and zero-point Energies= -1985.668979

Sum of electronic and thermal Energies= -1985.625206

Sum of electronic and thermal Enthalpies= -1985.624262

Sum of electronic and thermal Free Energies= -1985.758345

12 Triplet TX + methyl linoleate : transition state

Center Number	Atomic Number	Atomic Type	Coordinates (Angstroms) X	Y	Z
1	6	0	-1.479099	-0.428389	-2.893814
2	6	0	-0.015156	-0.857696	-3.122809
3	6	0	0.969761	0.108055	-2.532065
4	6	0	1.894217	-0.194063	-1.601467
5	6	0	2.832600	0.780964	-0.994459
6	6	0	4.188221	0.275738	-0.652618
7	6	0	5.319191	0.997506	-0.723717
8	6	0	6.688421	0.499774	-0.360223
9	6	0	7.678277	0.524118	-1.542539
10	1	0	-1.659866	-0.332452	-1.815761
11	1	0	-1.631639	0.574191	-3.319200
12	1	0	0.148056	-1.859229	-2.703440
13	1	0	0.157349	-0.940090	-4.207881
14	1	0	0.908367	1.137082	-2.892576
15	1	0	1.952668	-1.219214	-1.231816
16	1	0	2.868192	1.725680	-1.550829
17	1	0	4.240107	-0.749092	-0.280819
18	1	0	5.263338	2.025977	-1.086994
19	1	0	6.619294	-0.519682	0.041925
20	1	0	7.097499	1.127348	0.447633
21	1	0	7.290146	-0.115923	-2.346628
22	1	0	7.723266	1.541972	-1.956331
23	1	0	2.346237	1.095797	0.031205
24	8	0	1.605163	1.492744	1.355234
25	6	0	0.722828	0.521145	1.594646
26	6	0	1.188093	-0.690839	2.209803
27	6	0	2.557124	-0.819589	2.571814
28	6	0	3.045550	-1.973066	3.160604
29	6	0	2.195540	-3.060647	3.424203
30	6	0	0.845246	-2.965619	3.096117
31	6	0	0.342277	-1.804550	2.501488
32	6	0	-1.697772	-0.188047	1.476896
33	6	0	-3.026985	0.106919	1.157098
34	6	0	-3.361285	1.343670	0.609934
35	6	0	-2.348331	2.291031	0.376220

36	6	0	-1.031277	2.010019	0.689628
37	6	0	-0.651018	0.761510	1.256454
38	1	0	3.202280	0.029553	2.379894
39	1	0	4.097193	-2.033017	3.429313
40	1	0	2.579859	-3.964580	3.886794
41	1	0	0.173239	-3.794644	3.304210
42	1	0	-3.799939	-0.634391	1.344610
43	1	0	-4.395754	1.569007	0.369303
44	1	0	-0.244458	2.736025	0.524135
45	16	0	-1.381628	-1.799024	2.121792
46	6	0	-2.494815	-1.401729	-3.504269
47	1	0	-2.303159	-1.505056	-4.583037
48	1	0	-2.344406	-2.402287	-3.071968
49	6	0	-3.951272	-0.971276	-3.286561
50	1	0	-4.106123	0.019699	-3.736679
51	1	0	-4.132153	-0.845917	-2.209831
52	6	0	-4.966868	-1.960354	-3.867610
53	1	0	-4.861894	-2.951506	-3.409204
54	1	0	-5.996180	-1.623228	-3.699334
55	1	0	-4.829052	-2.081219	-4.949285
56	6	0	9.089914	0.068495	-1.152705
57	1	0	9.470886	0.716457	-0.349297
58	1	0	9.039972	-0.944817	-0.727124
59	6	0	10.080505	0.076526	-2.324207
60	1	0	10.128095	1.087991	-2.753687
61	1	0	9.702372	-0.575567	-3.125035
62	6	0	11.493444	-0.372923	-1.930308
63	1	0	11.872981	0.282707	-1.132307
64	1	0	11.444967	-1.382358	-1.494916
65	6	0	12.478616	-0.371279	-3.105579
66	1	0	12.531986	0.631696	-3.547420
67	1	0	12.111648	-1.029166	-3.903073
68	6	0	13.884690	-0.814916	-2.697412
69	1	0	14.295774	-0.164749	-1.913571
70	1	0	13.870717	-1.823127	-2.260873
71	6	0	14.865146	-0.827131	-3.854225
72	8	0	14.605240	-0.560409	-5.007556
73	8	0	16.101762	-1.184725	-3.433618

74	6	0	17.108863	-1.233053	-4.456668
75	1	0	17.231388	-0.252076	-4.923874
76	1	0	18.026934	-1.533256	-3.950356
77	1	0	16.840599	-1.960152	-5.228036
78	1	0	-2.602759	3.256637	-0.053212

Energy：

Zero-point correction= 0.666256 (Hartree/Particle)

Thermal correction to Energy= 0.705585

Thermal correction to Enthalpy= 0.706529

Thermal correction to Gibbs Free Energy= 0.582230

Sum of electronic and zero-point Energies= -1867.808045

Sum of electronic and thermal Energies= -1867.768715

Sum of electronic and thermal Enthalpies= -1867.767771

Sum of electronic and thermal Free Energies= -1867.892070

13 Methyl oleate + methyl oleate radical : transition state

Center Number	Atomic Number	Atomic Type	Coordinates (Angstroms) X	Y	Z
1	6	0	2.088030	6.737984	9.045788
2	6	0	2.122487	7.650314	7.835141
3	6	0	2.688203	6.971669	6.586216
4	6	0	2.723319	7.903647	5.368807
5	6	0	3.283286	7.230038	4.109428
6	6	0	3.335475	8.158654	2.889321
7	6	0	3.886787	7.469459	1.627589
8	6	0	3.976615	8.386829	0.417812
9	6	0	2.742196	8.742300	-0.273906
10	6	0	2.510122	9.890823	-0.948471
11	6	0	1.256894	10.201879	-1.716037
12	6	0	0.517494	11.460417	-1.218880
13	6	0	-0.733024	11.782401	-2.046156
14	6	0	-1.478222	13.039810	-1.579286
15	6	0	-2.728452	13.349405	-2.412640
16	6	0	-3.475179	14.610896	-1.960092
17	6	0	-4.725520	14.914976	-2.795145

18	6	0	-5.464279	16.178314	-2.341489
19	8	0	2.459173	5.584649	9.076803
20	8	0	1.577821	7.384072	10.121478
21	1	0	1.101295	8.015606	7.660647
22	1	0	2.707343	8.540951	8.102757
23	1	0	3.698313	6.602939	6.803971
24	1	0	2.088827	6.081235	6.358577
25	1	0	1.708608	8.274923	5.160957
26	1	0	3.327814	8.792360	5.604856
27	1	0	4.293867	6.850129	4.320078
28	1	0	2.672346	6.347149	3.869470
29	1	0	2.330360	8.549601	2.677782
30	1	0	3.958798	9.034300	3.122978
31	1	0	4.882556	7.059512	1.845613
32	1	0	3.248580	6.607382	1.381678
33	1	0	4.653902	9.235782	0.567623
34	1	0	4.736134	7.646026	-0.447314
35	1	0	1.958382	7.979695	-0.278601
36	1	0	3.280333	10.665460	-0.946074
37	1	0	0.577576	9.339253	-1.677581
38	1	0	1.504848	10.349680	-2.780149
39	1	0	1.204916	12.318669	-1.244393
40	1	0	0.240900	11.321941	-0.164545
41	1	0	-1.418607	10.922419	-2.015072
42	1	0	-0.446952	11.902207	-3.101884
43	1	0	-0.795042	13.901490	-1.614671
44	1	0	-1.764399	12.923170	-0.523463
45	1	0	-3.412774	12.488800	-2.372289
46	1	0	-2.441510	13.457830	-3.469284
47	1	0	-2.792597	15.472938	-2.002993
48	1	0	-3.762518	14.505098	-0.903182
49	1	0	-5.408915	14.055017	-2.749400
50	1	0	-4.438666	15.018027	-3.851415
51	1	0	-6.353305	16.365148	-2.954761
52	1	0	-4.818680	17.062440	-2.413028
53	1	0	-5.791607	16.092956	-1.297845
54	6	0	1.500114	6.605465	11.325854
55	1	0	1.071020	7.269411	12.077144

56	1	0	0.862361	5.729553	11.178539
57	1	0	2.494381	6.269903	11.633376
58	6	0	3.538304	3.636533	-9.427549
59	6	0	4.530420	4.046730	-8.356737
60	6	0	3.860803	4.592909	-7.094220
61	6	0	4.872113	5.014331	-6.021182
62	6	0	4.210508	5.557303	-4.747969
63	6	0	5.216576	5.984433	-3.671414
64	6	0	4.546906	6.522663	-2.393395
65	6	0	5.538158	6.956062	-1.323964
66	6	0	6.233337	5.915625	-0.574436
67	6	0	7.480440	5.999444	-0.058918
68	6	0	8.160523	4.916095	0.729427
69	6	0	9.407086	4.342263	0.025004
70	6	0	10.105932	3.241021	0.832443
71	6	0	11.333572	2.651248	0.126220
72	6	0	12.018071	1.530559	0.919799
73	6	0	13.238786	0.932829	0.208681
74	6	0	13.916556	-0.196117	0.996076
75	6	0	15.131478	-0.790689	0.276553
76	8	0	2.332403	3.731417	-9.351919
77	8	0	4.178218	3.138137	-10.512447
78	1	0	5.159241	3.175616	-8.128487
79	1	0	5.210888	4.788459	-8.797059
80	1	0	3.225191	5.445229	-7.364964
81	1	0	3.181625	3.831865	-6.689725
82	1	0	5.510217	4.156720	-5.760344
83	1	0	5.547530	5.778869	-6.433734
84	1	0	3.570048	6.412818	-5.008550
85	1	0	3.538054	4.790986	-4.334663
86	1	0	5.861763	5.134098	-3.409630
87	1	0	5.884174	6.756967	-4.080529
88	1	0	3.898383	7.369932	-2.654437
89	1	0	3.885089	5.746356	-1.981008
90	1	0	6.192104	7.774698	-1.646500
91	1	0	5.666759	4.998164	-0.392017
92	1	0	8.064452	6.904219	-0.241769
93	1	0	7.449046	4.103059	0.928813

94	1	0	8.467672	5.306629	1.713000
95	1	0	10.118097	5.156851	-0.176336
96	1	0	9.109800	3.947923	-0.956569
97	1	0	9.386739	2.435164	1.042018
98	1	0	10.405889	3.641373	1.812391
99	1	0	12.060414	3.453121	-0.071550
100	1	0	11.033222	2.265343	-0.859305
101	1	0	11.288294	0.731983	1.120451
102	1	0	12.323278	1.915733	1.904170
103	1	0	13.972756	1.728934	0.012853
104	1	0	12.934397	0.552985	-0.778192
105	1	0	13.182464	-0.990554	1.192590
106	1	0	14.223585	0.183208	1.981208
107	1	0	15.591694	-1.593936	0.863362
108	1	0	15.898991	-0.027560	0.097191
109	1	0	14.849334	-1.209454	-0.697420
110	6	0	3.325704	2.722321	-11.590872
111	1	0	3.995306	2.353800	-12.368820
112	1	0	2.646479	1.930778	-11.262546
113	1	0	2.733663	3.563805	-11.961122

Energy：

Zero-point correction= 1.031532 (Hartree/Particle)

Thermal correction to Energy= 1.087153

Thermal correction to Enthalpy= 1.088097

Thermal correction to Gibbs Free Energy= 0.922044

Sum of electronic and zero-point Energies= -1790.650417

Sum of electronic and thermal Energies= -1790.594796

Sum of electronic and thermal Enthalpies= -1790.593852

Sum of electronic and thermal Free Energies= -1790.759905

14 Methyl linoleate + methyl linoleate radical : transition state

Center Number	Atomic Number	Atomic Type	Coordinates (Angstroms) X	Y	Z
1	6	0	0.826229	-2.707111	1.280541
2	6	0	-0.269729	-1.925569	1.210841

3	6	0	-0.699449	-1.169510	0.032959
4	6	0	-2.096785	-0.747471	-0.066957
5	6	0	-2.746196	-0.456357	-1.212698
6	6	0	-4.171317	0.010486	-1.305169
7	6	0	1.276721	-3.453453	2.504342
8	1	0	1.434540	-2.839491	0.383532
9	1	0	-0.871659	-1.794629	2.112844
10	1	0	0.010081	0.004195	0.128109
11	1	0	-0.255322	-1.506956	-0.909087
12	1	0	-2.630010	-0.621303	0.877686
13	1	0	-2.220615	-0.585641	-2.161663
14	1	0	-4.206838	0.994237	-1.801028
15	1	0	-4.578384	0.159091	-0.295745
16	1	0	0.616267	-3.212465	3.348560
17	1	0	2.285216	-3.116356	2.794323
18	6	0	2.769897	0.469129	-1.203945
19	6	0	2.117760	0.756058	-0.058693
20	6	0	0.720260	1.178028	0.039273
21	6	0	0.287624	1.929761	1.218853
22	6	0	-0.807631	2.712325	1.288076
23	6	0	-1.261531	3.454214	2.513324
24	6	0	4.195289	0.002799	-1.294838
25	1	0	2.246586	0.601981	-2.153675
26	1	0	2.648694	0.626396	0.886764
27	1	0	0.278731	1.519265	-0.902629
28	1	0	0.886466	1.794466	2.122269
29	1	0	-1.412671	2.849161	0.389521
30	1	0	-0.604988	3.208285	3.359163
31	1	0	-2.271851	3.117819	2.797695
32	1	0	4.599977	-0.149763	-0.285047
33	1	0	4.232169	-0.978946	-1.794552
34	6	0	1.317959	-4.981442	2.302189
35	1	0	0.317518	-5.329664	2.010655
36	1	0	1.980880	-5.215961	1.456520
37	6	0	1.788304	-5.745440	3.546228
38	1	0	2.781550	-5.378362	3.845498
39	1	0	1.115549	-5.518367	4.386700
40	6	0	1.849576	-7.264691	3.342122

41	1	0	2.537157	-7.492907	2.514524
42	1	0	0.861731	-7.629056	3.024016
43	6	0	2.291652	-8.033728	4.593839
44	1	0	3.275653	-7.663851	4.919317
45	1	0	1.597715	-7.812487	5.418639
46	6	0	2.362142	-9.550209	4.377162
47	1	0	3.061093	-9.780084	3.563398
48	1	0	1.385869	-9.926438	4.046471
49	6	0	2.791034	-10.308093	5.635051
50	1	0	3.772533	-9.964728	5.989843
51	1	0	2.099699	-10.120245	6.467626
52	6	0	2.873292	-11.808010	5.428507
53	8	0	2.654189	-12.394918	4.391034
54	8	0	3.233244	-12.433116	6.574986
55	6	0	3.343927	-13.862372	6.486668
56	1	0	4.100789	-14.146011	5.750186
57	1	0	3.635609	-14.196093	7.483119
58	1	0	2.387213	-14.306148	6.197926
59	6	0	-1.298932	4.983141	2.317620
60	1	0	-0.296338	5.330715	2.032787
61	1	0	-1.957002	5.222556	1.469535
62	6	0	-1.774373	5.742696	3.562455
63	1	0	-2.770050	5.376497	3.854628
64	1	0	-1.106743	5.510497	4.405612
65	6	0	-1.831186	7.262944	3.364618
66	1	0	-2.513367	7.496214	2.533964
67	1	0	-0.840687	7.626506	3.053934
68	6	0	-2.278971	8.027591	4.617004
69	1	0	-3.265695	7.658514	4.935061
70	1	0	-1.590409	7.801316	5.444937
71	6	0	-2.344861	9.545126	4.406382
72	1	0	-3.038658	9.780067	3.589677
73	1	0	-1.365858	9.920506	4.082839
74	6	0	-2.779112	10.298702	5.665022
75	1	0	-3.763214	9.955763	6.013023
76	1	0	-2.092740	10.106216	6.500612
77	6	0	-2.857684	11.799578	5.464050
78	8	0	-2.635120	12.389969	4.429292

79	8	0	-3.218901	12.421077	6.612092
80	6	0	-3.326810	13.850848	6.528828
81	1	0	-4.082234	14.138559	5.792442
82	1	0	-3.619133	14.181497	7.526115
83	1	0	-2.368921	14.293873	6.242863
84	6	0	-5.080093	-0.957470	-2.089237
85	1	0	-4.651344	-1.127014	-3.087861
86	1	0	-5.077785	-1.934696	-1.586986
87	6	0	-6.521132	-0.452566	-2.233418
88	1	0	-6.947371	-0.277346	-1.234152
89	1	0	-6.514644	0.526717	-2.735522
90	6	0	-7.432129	-1.412498	-3.009286
91	1	0	-7.439377	-2.390030	-2.506519
92	1	0	-7.006507	-1.588065	-4.007582
93	6	0	-8.869383	-0.900483	-3.150003
94	1	0	-8.898341	0.058622	-3.681858
95	1	0	-9.494500	-1.608406	-3.706231
96	1	0	-9.333241	-0.746885	-2.167759
97	6	0	5.105694	0.974081	-2.072886
98	1	0	4.679290	1.147412	-3.071867
99	1	0	5.101844	1.949326	-1.566811
100	6	0	6.547251	0.470248	-2.215601
101	1	0	6.971121	0.291086	-1.216025
102	1	0	6.542349	-0.506965	-2.721738
103	6	0	7.459761	1.433715	-2.985285
104	1	0	7.465377	2.409169	-2.478478
105	1	0	7.036504	1.613229	-3.983886
106	6	0	8.897571	0.922883	-3.124606
107	1	0	8.928255	-0.034000	-3.660350
108	1	0	9.523734	1.633369	-3.676372
109	1	0	9.359102	0.765428	-2.141876

Energy：

Zero-point correction= 0.983090 (Hartree/Particle)

Thermal correction to Energy= 1.038114

Thermal correction to Enthalpy= 1.039058

Thermal correction to Gibbs Free Energy= 0.871441

Sum of electronic and zero-point Energies= -1788.250344

Sum of electronic and thermal Energies= -1788.195321
Sum of electronic and thermal Enthalpies= -1788.194377
Sum of electronic and thermal Free Energies= -1788.361993

vertical excitation energies M062x functional with 6-31g(d) basic set

ITX

Excited State 1: Singlet-A 3.7708 eV 328.80 nm f=0.0000 <S**2>=0.000 (=86.96 kcal/mol)

TX

Excited State 1: Singlet-A 3.7143 eV 333.80 nm f=0.0000 <S**2>=0.000 (=85.65 kcal/mol)
